# Adaptive Glomerular Hypertrophy in Kidney Transplants Associated With Focal Segmental Glomerulosclerosis: Report of Three Cases

**DOI:** 10.1155/crin/7613213

**Published:** 2025-12-29

**Authors:** Autumn LaRocque, Ujwal Gautam, Nora Ewis, Richard Ugarte, Emily Daniel, John C. Papadimitriou, Abdolreza Haririan, Cinthia B. Drachenberg

**Affiliations:** ^1^ Department of Pathology, University of Maryland School of Medicine, Baltimore, Maryland, USA, umaryland.edu; ^2^ Department of Medicine, University of Maryland School of Medicine, Baltimore, Maryland, USA, umaryland.edu

## Abstract

Transplant kidneys face increased functional demands in comparison to physiological conditions, and glomerular hypertrophy (GH) appears to be a common compensatory mechanism. Adaptive GH could contribute to improved graft function, but a so‐called “maladaptive” response may lead to shortened graft survival. We assessed in three patients the mean glomerular diameter in donor preimplantation wedge biopsies and in subsequent posttransplant biopsies and correlated the glomerular size with Banff scores and clinical course. Mean glomerular size increased from 192.09 ± 28.65, 188.98 ± 19.86, and 168.96 ± 18.5 in preimplantation biopsies to 279.43 ± 50.6 (*p* < 0.0001), 275.23 ± 68.17 (*p* < 0.0001), and 266.23 ± 40.5 (*p* < 0.00001) in the last biopsies, respectively. The proportion of enlarged glomeruli (> 200 µ) increased from 42.86%, 31%, and 5% in the donor biopsies to 93.3% (*p*0.008), 95.7% (*p* < 0.0001), and 95.1% (*p* < 0.00001) in the last biopsy, respectively. Although GH was initially associated with the achievement of good graft function, secondary FSGS and proteinuria developed at 3, 7, and > 17 years posttransplant, respectively. GH (glomerulomegaly) can be easily appreciated by light microscopy, but is not routinely recorded in transplant biopsy reports. Recognition and documentation of GH could help identify factors associated with the “maladaptive” phase of this adaptive response and find interventions that can potentially prolong graft survival.

## 1. Introduction

Reduction of renal mass or disproportion between nephron endowment and body mass leads to compensatory hyperfiltration and glomerular enlargement/hypertrophy (GH) [1]. This adaptive process and its clinical and morphological features were described in native kidney biopsies from patients with obesity related glomerulopathy (ORG) that is characterized histologically by glomerulomegaly with eventual development of FSGS in hypertrophic glomeruli [2–4].

In the groundbreaking study of ORG, mean glomerular size was 1.34 times larger than controls (226 vs. 169 μm) [3].

Hypertrophy of the remaining kidney has also been documented in the setting of living kidney donation [5]. The adaptive changes after donor nephrectomy are associated with increased risk for proteinuria. The dynamic nature of the compensatory process is highlighted by a recent study demonstrating that proteinuria is more likely to occur after renal donation in donors with kidneys that were already enlarged at the time of transplantation and have less reserve for potential adaptation [6].

In histological and ultrasonic studies, the average diameter of normal glomeruli ranges from 167 to 200 microns (μm) [3, 7, 8]. In native kidneys, a glomerular diameter of ≥ 224 μm was associated with FSGS and worse prognosis in IgA nephropathy [9, 10].

Glomerular size can be assessed on light microscopy with available software [9], but glomerular measurements are not routinely included in biopsy reports. In general, if glomerulomegaly is suspected, the glomerular diameter is estimated by comparison to the diameter of the field of view at 400x magnification (10x ocular lens and 40x objective lens), which typically measures 500 μm.

After renal transplantation, adaptive GH is expected to occur in the transplanted organ, but the long‐term impact of this process is obscured by the multiplicity of immunological and nonimmunological factors affecting graft outcomes [9, 11]. The size of the nephron mass at the time of transplantation, donor body mass index, and the donor–recipient size relationship were found to impact glomerular size, particularly in the first‐year posttransplantation, but these parameters are not measured routinely in practice [12–17].

We present three patients who never experienced rejection and had stable graft function after transplantation, but developed GH in subsequent biopsies, and eventually FSGS. Glomerular measurements were correlated with the main Banff scores and the clinical course. Electron microscopy was performed on all samples. Material for immunofluorescence studies was only available for the biopsies obtained for proteinuria (Table 1).

**Table 1:** Pathology and clinical data at the time of preimplantation donor biopsy and subsequent allograft biopsies.

Pathology and clinical data	Donor biopsy	First biopsy	Second biopsy	Last biopsy
*Patient 1*	Pretransplant wedge biopsy	3‐Month surveillance biopsy	6‐Month surveillance biopsy	18‐Month surveillance biopsy
% Glomerulosclerosis (FSGS)	16.4% (none)	16.6% (none)	0% (none)	0% (1 perihilar FSGS lesion)
IFTA	1 (15%)	1 (10%)	0 (< 5%)	0% (≈5%)
Vascular sclerosis	1	1	1	1
Mean glomerular diameter	192.09 ± 28.65	176.03 ± 16.43, *p* < 0.0001^^∗^	242.2 ± 36.9, *p* < 0.0001^^^	279.43 ± 50.6, *p* < 0.0001^^#^
Median glomerular diameter	192.8	175.6	245.3	283.2
% Glomeruli > 200 µ in diameter	42.86%	7.2%, *p* < 0.00001^^∗^	85.1%, *p* < 0.00001^^^	93.3%, *p* < 0.00001^^^, *p*0.008^#^
# of glomeruli measured	217	66	102	358
Serum creatinine (SCr)	Unknown	2.08 mg/dL (45 mil/min)	1.67 mg/dL (49 mil/min)	1.45 mg/dL (58 mil/min)
Proteinuria	Unknown	No	MACR 196 mcg/mgC	MACR 69.8 mcg/mgC
Electron microscopy	Normal	Normal	Normal	Normal except for the FSGS area
BMI recipient (BMI donor)	28.9 (22)	28.1	26.3	26.9

*Patient 2*	Pretransplant wedge biopsy	3‐Week biopsy (laparotomy)	12‐Month surveillance biopsy	8 y Biopsy, ↑ SCr, proteinuria^++^
% Glomerulosclerosis (FSGS)	0%	0%,	0%	16% (1 FSGS lesion)
IFTA	0 (≈5%)	1 (20%)	1 (20%)	1 (20%)
Vascular sclerosis	1	2	2	2
Mean glomerular diameter	188.98 ± 19.86	187.22 ± 23.93 NS^^^	217.01 ± 30.6, *p* < 0.0001^^#^	275.23 ± 68.17, *p* < 0.0001^^#^
Median glomerular diameter	183.4	176.7	209.9	250.2
% Glomeruli > 200 µ in diameter	31%	35.8% NS^	74.2%, *p* < 0.00001^^#^	95.7%, *p* < 0.00001^^#^
# of glomeruli measured	157	168	92	104
Serum creatinine	Unknown	2.96 mg/dL (48 mil/min)	1.26 mg/dL (55 mil/min)	4.3 mg/dL (12 mil/min)
Proteinuria	Unknown	No	MACR 32 mcg/mgC	MACR 855 mcg/mgC
Electron microscopy	Normal	Normal	Normal	Effacement ≈ 50% patent loops
BMI recipient (BMI donor)	44.3 (27.3)	42.9	37.8	35.5

*Patient 3*	Pretransplant wedge biopsy	2‐Week biopsy for DGF (HD)		7y Biopsy, heavy proteinuria^++^
% glomerulosclerosis (FSGS)	0%	0%		10% (2 FSGS lesions)
IFTA	0 (≈5%)	1 (≈10%)		1 (≈15%)
Vascular sclerosis	0–1	0		2
Mean glomerular diameter	168.96 ± 18.5	193.04 ± 23.7 NS^^^		266.23 ± 40.5 *p* < 0.00001^^#^
Median glomerular diameter	165.4	192.6		272.4
% Glomeruli > 200 µ in diameter	5%	5.8% NS^		95.1% *p* < 0.00001^^#^
# of glomeruli measured	188	59		79
Serum creatinine (eGFR)	Unknown	N/A		1.41 mg/dL (45 mL/min)
Proteinuria	Unknown	No		MACR 4356 mcg/mgC
Foot processes of podocytes (EM)	Normal	Unremarkable		Effacement ≈ 15% patent loops
BMI recipient (BMI donor)	24.3 (unknown)	26.3		23.4

Abbreviations: FSGS, focal segmental glomerulosclerosis; HD, hemodialysis.

^^^Comparison with the donor biopsy (*z* score for 2 population proportions).

^#^Comparison with the previous biopsy (*z* score for 2 population proportions).

^∗^Values appear inconsistent with findings in the donor and subsequent biopsies. This may be related to a tissue sampling variation.

^++^Immunofluorescence (IF) studies for IgG, IgA, IgM, C3, C1q, albumin, fibrinogen, and kappa and lambda light chains, with the following results. Patient 2: the IF sections contained 2 globally sclerosed and 2 normal appearing glomeruli. Immunostains were negative in the patent glomeruli, and specks of C3 positivity were noted in the sclerotic glomeruli. Patient 3: the IF sections contained 3 patent glomeruli, one with a segmental sclerotic lesion, and 1 globally sclerosed glomerulus. The C3 and IgM stains weakly marked the segmental sclerotic lesion. No other glomerular staining was noted. Stains for C3 highlighted reabsorption tubular droplets in clusters of proximal tubules.

To assess glomerular size, the largest diameter in nonsclerosed glomeruli was measured in 3 sections from each donor wedge (175–217 glomeruli) and 11 sections from each posttransplant biopsy (66–358 glomeruli) (Table 1). Glomeruli ≤ 140 µ were excluded in order to avoid the wide variation of incomplete/tangential glomerular cuts. Mean glomerular diameters and percentage of glomeruli > 200 μm were compared using *t* and *z* tests, respectively.

## 2. Case Reports

**Figure 1:**
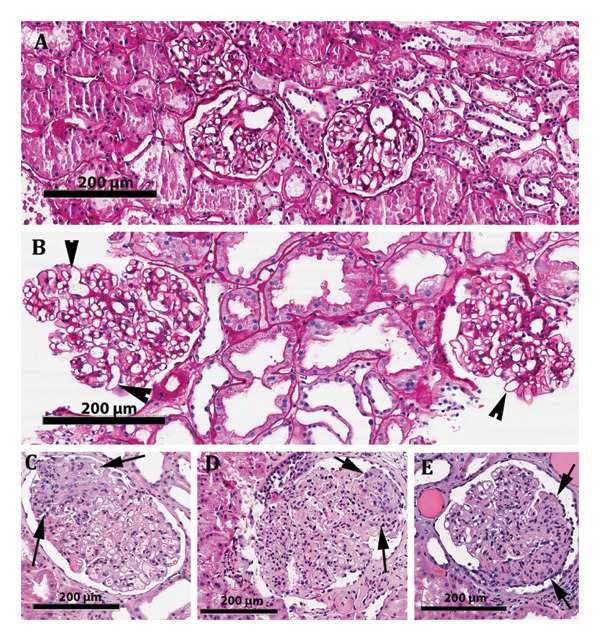
Light microscopy images. (A) Representative image of 3‐month surveillance biopsy from Patient 1, with normal appearing glomeruli in a background of normal tubulointerstitium. (B) Representative image of the 18‐month surveillance biopsy from Patient 1 showing significant GH in comparison to the previous biopsy. Occasional enlarged and irregularly shaped capillary loops are characteristic of GH (arrowheads). There is no evidence of acute rejection. Mild arteriolar hyalinosis and some tubular injury are noted. (C–E) Images from the last biopsy of Patients 1, 2 and 3, demonstrating their respective focal segmental areas of glomerulosclerosis highlighted by arrows. There were no features of acute rejection. The remaining glomeruli were enlarged but otherwise normal on light microscopy.

Patient 1 is a 51‐year‐old man with a BMI of 28.9 and a history of hypertension. The donor, a 52‐year‐old woman with drug overdose anoxia and a BMI of 22, had no other comorbidities. The posttransplant course was complicated by delayed graft function (DGF), posttransplant diabetes, and erythrocytosis. There was early CMV viremia and low‐level Class I donor‐specific antibody (DSA). Surveillance biopsies at 6 and 18 months were negative for rejection and showed progressive GH and development of FSGS in the second biopsy (Figures 1(A), 1(B), and 1(C)). Graft function continued to improve until the last follow‐up 36 months posttransplant (Scr: 1.41 mg/dL, eGFR: 59).

Patient 2 is a 57‐year‐old woman with a history of ADPKD and hypertension, who received a kidney transplant 8 years earlier, from a deceased donor kidney from a 52‐year‐old with a kidney donor profile index of 71% and a BMI of 27.3. The posttransplant course was complicated by DGF, CMV viremia, and leucopenia. There was never rejection or the presence of DSA. The patient was obese at transplantation, with a BMI of 44.3. After transplantation, nutritional interventions resulted in progressive BMI reduction to 35.5 at the time of the last biopsy (Table 1). On a 12‐month surveillance biopsy, there was GH in comparison to the preimplantation and the 3‐week posttransplant biopsies. Graft function was stable (Scr: 1.2–1.3 mg/dL, eGFR: 41–55) until the 7th year posttransplant. A biopsy prompted by worsening creatinine and proteinuria on the 8^th^ year posttransplantation showed further increase in GH and the presence of FSGS (Figure 1(D)). Graft function continued deteriorating, and the patient was listed for retransplantation.

Patient 3 was a 66‐year‐old with a history of hypertension and diabetes mellitus, when he received a deceased donor kidney from a 28‐year‐old male with an unknown history. The posttransplant course was complicated by DGF and resections of a native renal cell and colon carcinomas. The BMI was stable (23–26), and diabetes was well controlled (HBA1c: 7%–7.6%). The patient never experienced rejection or DSA and had stable graft function (Scr:1.2–1.4 mg/dL, eGFR: up to 65). The donor wedge biopsy, as well as an early posttransplant biopsy performed for DGF, demonstrated a good‐quality kidney with insignificant glomerulosclerosis and lack of fibrosis and vascular sclerosis. The glomeruli had a normal appearance and size. At the 17^th^ year, a biopsy for nephrotic range proteinuria showed prominent GH in comparison to the previous biopsies, as well as FSGS (Figure 1(E); Table 1). The patient died with a functioning graft, 13 months later, due to intestinal necrosis.

All three patients received alemtuzumab induction with steroid tape and maintenance immunosuppression including tacrolimus, mycophenolate sodium (MPA), and prednisone for Patient 1 and tacrolimus with MP for Patients 2 and 3.

A summary of morphological and clinical findings including electron microscopy and immunofluorescence results is presented in Table 1.

All the patients allowed personal data processing, and informed consent was obtained from all individual participants included in the study.

## 3. Discussion

Hyperfiltration and GH are dynamic processes, occurring in a variety of physiological and pathological conditions. Before transplantation, the estimated functional donor kidney mass may be considered to select the most appropriate recipient and minimize hyperfiltration [13]. Nevertheless, most kidney transplants undergo adaptive changes, including GH, to adjust to the increased functional demands. Comparisons between implantation and 12‐month protocol biopsies show a universal increase in glomerular size over time [18, 19]. Regarding donor factors, posttransplant biopsies of kidneys from high BMI donors showed GH that did not affect short‐term outcomes [17]. On the other hand, larger glomerular size/volume in preimplantation, implantation, and early biopsies, presumably related to donor‐related factors, correlate with worse graft outcomes [14, 15, 20, 21]. In addition, body size mismatch between donor and recipient, calcineurin inhibitor toxicity, and other posttransplant processes such as acute and chronic rejection can impact glomerular size [9, 11].

A large study of for‐cause biopsies from a prospective cohort of grafts with good initial function compared to a cross‐sectional cohort with late graft dysfunction suggested that glomerular size generally increases with time, and that GH is a multifaceted process that may relate to a variety of other histopathological abnormalities. More specifically, larger glomeruli were found in association with FSGS, interstitial fibrosis, arterial sclerosis, and glomerulitis/transplant glomerulopathy [9].

Of interest, for grafts biopsied later (mean 7.7 years posttransplant), larger glomerular size correlated with better eGFR and better graft survival, provided there was no histological evidence of AMR or diabetic glomerulosclerosis [9]. This finding suggests that in the absence of other common glomerular pathologies, GH is beneficial.

Although biopsy evaluation provides specific information on the morphological status of the kidney, there are other methods that can assess global changes relating to the size of the organ. More specifically, imaging studies of the remaining kidney after living kidney donation have demonstrated a progressive increase in renocortical volume supporting ongoing hyperfiltration and nephron/GH [5, 6].

Renal allograft biopsies are routinely performed under ultrasound guidance, and ultrasound measurements of cortical thickness have shown correlation with eGFR [22]. Prospective studies of allograft cortical thickness measured with ultrasonography could provide information regarding the degree of cortical hypertrophy after transplantation and suggest the need for preventive interventions.

The three patients presented in this report lacked inflammatory features, indicating allograft rejection, and overall showed only mild chronic features. In these patients, development of progressive GH likely contributed to stable graft function for 3, > 7, and > 17 years, respectively, but their later biopsies displayed striking FSGS lesions, together with mild to marked proteinuria. Interestingly, the donor biopsy sample from the graft with the longest survival displayed glomeruli of normal size, in contrast to the other donors that had a significant proportion of enlarged glomeruli already at implantation (Table 1).

For Patient 2, obesity undoubtedly contributed to the burden of functional demands on the graft; despite some weight loss after transplantation, the BMI remained above 35.

In aggregate, the available data suggest that GH represents both a desirable adaptive response to increased renal functional needs, but may also be associated with chronic (progressive) parenchymal injury, and its presence may contribute to evolving renal failure [9, 10, 23].

In the long term, GH can lead to secondary FSGS by indirect injury to podocytes. In the early stages of hyperfiltration, the overall glomerular architecture is preserved, except for the appearance of enlarged tufts and some capillaries with unusual shapes and increased size [24].

**Figure 2:**
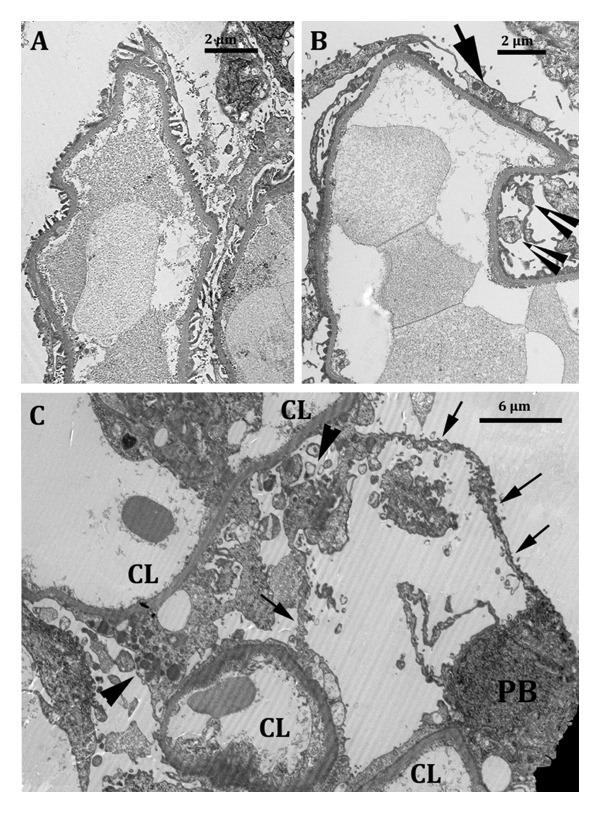
Electron microscopy images. (A) Six‐month surveillance biopsy from Patient 1 shows abnormally dilated capillary loops consistent with GH. The podocyte lining is normal with preserved foot processes of podocytes. (B) An eight‐year for‐cause biopsy from Patient 2 shows markedly dilated capillary loops in the patent glomeruli. The podocyte lining is abnormal with segments of effaced foot processes (arrow) and partially detached fragments of injured podocytes (arrowheads). FSGS was noted in one glomerulus (Figure 1(D)). (C) A seventeen‐year‐old biopsy from Patient 3 demonstrating patent capillary lumina (CL) in several loops. A podocyte body (PB) is noted on the bottom right, from which thin sheets of cytoplasmic fragments line an irregularly shaped pseudocystic space (arrows). Fragments of injured podocyte cytoplasm are noted between the arrowheads.

**Figure 3:**
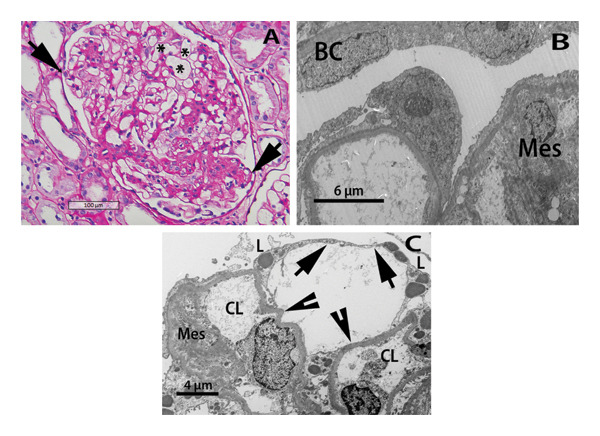
Light and electron microscopy images. (A) Hypertrophic glomerulus with irregular increase in mesangial matrix and areas of segmental sclerosis connected with the glomerular hilum. Occasional capillaries are enlarged and have anomalous shapes (asterisks). Arrows mark peripheral capillary loops in close proximity to the Bowman’s capsule. With progression of the podocyte injury, the latter areas are prone to form tuft adhesions(PAS stain). (B) Two peripheral capillary loops and an adjacent segment of the Bowman’s capsule (BC) are shown. The capillary on the left is partially covered by an enlarged podocyte body with elongated/flattened cytoplasmic processes toward the left. The loop on the right is partially solidified with abundant mesangial matrix (Mes) and complete fusion of the foot processes of podocytes. (C) Marked degenerative changes in the podocyte lining (arrows) have led to the formation of a pseudocyst linking two capillary loops (CL) with denuded basement membranes (arrowheads). There is a focal increase in mesangial matrix (Mes). Electron‐dense rounded structures most consistent with lysosomes (L) are noted in degenerated podocyte cytoplasm.

With progressive enlargement of capillary loops, there is relative or absolute podocyte depletion [25, 26]. The podocytes overlying the enlarged loops appear hypertrophic, and their cell bodies stretch in order to cover larger capillary surface areas [27]. With worsening podocyte injury, their cytoplasm becomes thin and attenuated (“pancake podocytes”), and large spaces (“pseudocysts”) develop below the podocyte body (Figures 2 and 3). The enlarged peripheral capillary loops, often with segments of naked basement membrane and large pseudocysts, tend to approximate the Bowman’s capsule, leading to the development of tuft adhesions and FSGS [3, 24, 27–29]. In the earlier stages of hyperfiltration, the mesangial matrix is unremarkable; however, irregular but often significant mesangial accentuation is seen, with the potential development of sclerotic lesions [24] (Figure 3).

Serial electron microscopic studies in animal models suggest that the successful “adaptive” phase of GH consists of increased glomerular size with good preservation of the podocyte lining. In contrast, irreversible podocyte injury, such as the formation of pseudocysts, naked segments of basement membrane with the tendency to form adhesions to the Bowman’s capsule, and FSGS lesions, characterizes the “maladaptive” phase of the process [24, 27].

Evaluation of subsequent biopsies in these three cases demonstrates that identification of glomerulomegaly in an otherwise normal biopsy is unlikely to predict “adaptive” vs. “maladaptive” GH. Because this is a dynamic compensatory process, development of proteinuria, appearance of FSGS, or progressive decrease in the eGFR are the features that may indicate, in practice, that the benefit of the compensatory process is reaching its limits [18, 29].

In the absence of the specific factors mentioned above (e.g., suboptimal donor kidney and recipient obesity), it is unclear when and what causes this adaptive response to become maladaptive. Nevertheless, active interventions in order to keep ideal control of body weight, blood pressure, and diabetes mellitus can minimize functional stress, limiting as much as possible worsening hyperfiltration leading to maladaptive GH and FSGS [30, 31].

In conclusion, these three cases exemplify the natural history of posttransplant compensatory GH, a process that likely contributes to the success of renal transplantation but could also lead to graft dysfunction. All three patients eventually developed FSGS lesions, which did not preclude the maintenance of stable graft function in Patients 1 and 3 but coincided with a precipitous decline of renal function and eventual failure in Patient 2. Even in its purest form, occurring independently of other pathologies, postadaptive GH still represents a poorly understood entity that reflects both adaptation and potential risk for graft deterioration. Our observations suggest that glomerular hypertrophy, in kidney transplant biopsies, may be a predictor of graft outcomes and deserves documentation and further investigation.

**Nomenclature**
ADPKD: Adult polycystic kidney disease
AMR: Antibody‐mediated allograft rejection
BMI: Body mass index
CMV: Cytomegalovirus
DSA: Donor‐specific antibody
DGF: Delayed graft function
eGFR: Estimated glomerular filtration rate
IFTA: Interstitial fibrosis/tubular atrophy
Scr: Serum creatinine

## Conflicts of Interest

The authors declare no conflicts of interest.

## Author Contributions

All authors contributed to data acquisition. Clinical data were obtained by Abdolreza Haririan, Ujwal Gautam, Richard Ugarte, and Emily Daniel. Pathology data and measurements were obtained by Autumn LaRocque, Nora Ewis, John C. Papadimitriou, and Cinthia B. Drachenberg. The article was drafted by Cinthia B. Drachenberg and was revised critically by all authors.

## Funding

Specific funding was not provided. This is authors’ own work.
